# Non-traumatic Rectus Sheath Hematoma During Direct Oral Anticoagulation

**DOI:** 10.7759/cureus.45876

**Published:** 2023-09-24

**Authors:** Takuto Hideyama, Eri Watanabe, Nobuhiro Ido, Hiroo Terashi, Hitoshi Aizawa

**Affiliations:** 1 Neurology, Tokyo Medical University Hospital, Tokyo, JPN

**Keywords:** carnett sign, fothergill sign, direct oral anticoagulants, abdominal pain, rectus sheath hematoma

## Abstract

We report a case of anticoagulation therapy complicated by a non-traumatic rectus sheath hematoma (RSH). RSH is a relatively rare occurrence caused by bleeding into the rectus sheath following the rupture of the superior and inferior epigastric vessels combined with a primary tear of the rectus muscle fibers. Herein, we report a rare presentation of RSH in a 73-year-old man taking the direct oral anticoagulant (DOAC) apixaban orally. The patient presented with sudden right abdominal pain after a severe cough, which worsened with cough and movement. The Fothergill and Carnett signs were positive. The platelet count, renal function test, and the prothrombin time/international normalized ratio were within the normal range. The activated partial thromboplastin time was 40.0 s, slightly longer than normal. Computed tomography (CT) of the abdomen and pelvis showed RSH, and DOAC therapy was temporarily discontinued. Subsequently, RSH resolution was confirmed via CT four weeks after the onset. DOACs are safer and more efficacious than warfarin for patients with non-valvular atrial fibrillation. However, RSH is a potential complication of anticoagulant therapy. This case report demonstrates that RSH should be considered in the differential diagnosis of sudden-onset abdominal pain and mass in patients on DOACs.

## Introduction

Non-traumatic rectus sheath hematoma (RSH) is a relatively rare occurrence caused by bleeding into the rectus sheath following rupture of the superior and inferior epigastric vessels, combined with a primary tear of the rectus muscle fibers [1]. Among older adults, the number of patients with non-valvular atrial fibrillation taking direct oral anticoagulants (DOACs) is increasing [2]. Although DOACs are safer than warfarin, they carry bleeding risks such as cerebral and gastrointestinal hemorrhage [3]. Here, we report a case of anticoagulation therapy complicated by a non-traumatic RSH. The presented clinical picture is simple yet noteworthy, as it involves a patient taking a DOAC.

## Case presentation

A 73-year-old man visited our department complaining of sudden right abdominal pain, which occurred after a severe cough due to allergic rhinitis. This pain worsened with coughing and movement. The patient was taking apixaban (5 mg, orally twice daily), a DOAC, for stroke prevention due to atrial fibrillation. Vital signs were within normal limits. The patient had no anemia of the eyelid conjunctiva and normal heart and lung sounds on chest auscultation. Skin findings were normal. Abdominal findings revealed a soft abdominal wall and no muscular defense. However, we observed positive Fothergill and Carnett signs, physical examination findings that can help distinguish intrabdominal from abdominal wall conditions such as RSH [4].

**Figure 1:**
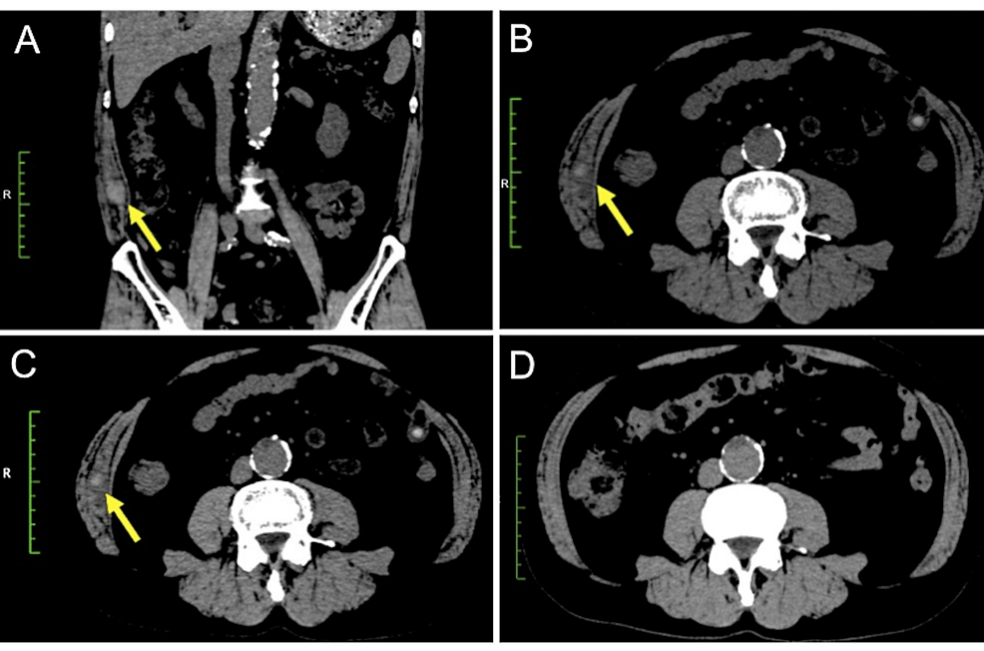
CT findings of the abdomen and pelvis (A, B) CT scan on Day 1 revealed a 22 × 18 × 10 mm right RSH with a high attenuation. RSH does not cross the midline or dissect fascial planes (arrow). (C) CT scan obtained one week after the onset of the illness revealed a reduced RSH size (12 × 11 × 8 mm). Abbreviations: CT, computed tomography; RSH, rectus sheath hematoma

The patient’s hemoglobin level, platelet count, renal function test results, and urinalysis results were normal. The prothrombin time/international normalized ratio was 1.22, and the activated partial thromboplastin time was 40.0 s, slightly longer than normal. Additionally, computed tomography (CT) of the abdomen and pelvis showed a 20 × 15 × 10 mm right RSH with a high-density area for subacute hematoma (Figures 1A, 1B).

DOAC therapy was temporarily discontinued. The reason was the risk of enlargement due to rebleeding for persistent coughing and the high density of the hematoma on CT. These findings led us to consider that the hematoma had not yet coagulated. A follow-up CT performed one week after the onset of the illness showed a reduction in the size of the RSH (Figure 1C). The patient was able to resume DOAC therapy. Subsequently, the RSH resolution was confirmed using CT imaging four weeks after the onset (Figure 1D).

## Discussion

RSH is a rare clinical condition caused by damage to the superior and inferior epigastric arteries. This can occur due to trauma, exercise [5,6], or severe coughing due to COVID-19 [7]. Pain associated with RSH is also typically characterized by worsening with movement [5,6]. In a previous report, 1.8% of 1,257 patients with abdominal pain were ultimately diagnosed with RSH [8]. Spontaneous RSH has been previously reported in association with warfarin administration for the treatment of thromboembolic pulmonary hypertension [9]. RSH is a complication of anticoagulant therapy. DOACs are generally safe and effective, with less bleeding risk than warfarin for patients with nonvalvular atrial fibrillation. Pain associated with RSH is also typically characterized by worsening with movement [5,6]. Fothergill's and Carnett's signs are physical examinations that can help distinguish whether the pain originates from the abdominal organs or the abdominal wall and can be used to diagnose RSH [4]. A CT scan is effective in confirming the diagnosis of RSH [5-7,9].

The patient denied any recent traumatic event or injury. We assumed that, in this case, coughing increased abdominal pressure, calcification of the rib cartilage caused intramuscular vascular strangulation, and the coagulopathy caused by the DOAC accelerated hematoma formation. Intramuscular hemorrhage while taking anticoagulant medication requires blood transfusion or embolization of the dominant vessels from the lumbar or inferior epigastric artery [6]. Therefore, we decided to prevent further bleeding by temporarily discontinuing the DOAC, which resulted in a good outcome. This case report demonstrates that RSH should be considered in the differential diagnosis of sudden-onset abdominal pain and mass when managing patients on DOACs.

## Conclusions

DOACs are safe and effective for patients with non-valvular atrial fibrillation. However, RSH is a potential complication of DOACs. This case emphasizes the importance of considering RSH in the differential diagnosis of sudden abdominal pain in patients on DOACs.
